# Supplementary material for: MutLα suppresses error-prone DNA mismatch repair and preferentially protects noncoding DNA from mutations
Source: J Biol Chem. 2024 May 21;300(6):107406. doi: 10.1016/j.jbc.2024.107406 (PMC11231602; doi:10.1016/j.jbc.2024.107406)
Supplement: Supporting Information [file mmc1.docx]

SUPPORTING INFORMATION

**MutLα suppresses error-prone mismatch repair and preferentially**

**protects noncoding DNA from mutations**

Lyudmila Y. Kadyrova ^1,*^, Piotr A. Mieczkowski ^2,*^, and Farid A. Kadyrov ^1,3^

^1^ Department of Biochemistry and Molecular Biology, Southern Illinois University School of Medicine, Carbondale, IL 62901, USA.

^2^ Department of Genetics, Lineberger Comprehensive Cancer Center, University

of North Carolina, Chapel Hill, North Carolina 27599, USA.

*These authors contributed equally to this study.

^3^ To whom correspondence may be addressed: [fkadyrov@siumed.edu](mailto:fkadyrov@siumed.edu)

Running title: MutLα and genome-wide MMR

MATERIALS INCLUDED:

TABLES S1-S3

**Table S1. Mutation spectra in the *wild-type*, *pms1Δ*, and *msh2Δ* diploid yeast strains**

|  | ***wild-type*** | ***pms1Δ*** | ***msh2Δ*** |
| --- | --- | --- | --- |
| **Isolates** | 15 | 9 | 8 |
| **Passages** | 450 | 270 | 240 |
| **Generations** | 13,500 | 8,100 | 7,200 |
| **Genome size** | 22,983,805 bp | | |
| **Mutation type** | **Number of mutations in each category (n)** | | |
| **Deletions of single A/T pairs** | 0 | 2,008 | 1,572 |
| **Deletions of single G/C pairs** | 0 | 73 | 68 |
| **Insertions of single A/T pairs** | 1 | 217 | 154 |
| **Insertions of single G/C pairs** | 0 | 17 | 13 |
| **>1-bp deletions** | 0 | 843 | 611 |
| **>1-bp insertions** | 0 | 65 | 42 |
| **T→C** | 6 | 213 | 177 |
| **C→T** | 10 | 316 | 352 |
| **T→A** | 3 | 28 | 27 |
| **T→G** | 5 | 24 | 18 |
| **C→A** | 19 | 144 | 154 |
| **C→G** | 6 | 9 | 13 |
| **other** | 1 | 4 | 3 |
| **Total** | 51 | 3961 | 3,204 |

**Table S2. Mutation rates in the *pms1Δ*, *msh2Δ*, and *wild-type* diploid yeast strains**

|  | ***wild-type*** | ***pms1Δ*** | ***msh2Δ*** |
| --- | --- | --- | --- |
| **Isolates** | 15 | 9 | 8 |
| **Passages** | 450 | 270 | 240 |
| **Generations** | 13,500 | 8,100 | 7,200 |
| **Genome size** | 22,983,805 bp | | |
| **Mutation type** | **Absolute rate in each category (x 10^-11^)** | | |
| **Deletions of single A/T pairs** | < 0.32 | 1,078 | 950 |
| **Deletions of single G/C pairs** | < 0.32 | 39 | 41 |
| **Insertions of single A/T pairs** | 0.32 | 117 | 93 |
| **Insertions of single G/C pairs** | < 0.32 | 9 | 8 |
| **>1-bp deletions** | < 0.32 | 453 | 369 |
| **>1-bp insertions** | < 0.32 | 35 | 25 |
| **T→C** | 1.9 | 115 | 108 |
| **C→T** | 3.2 | 170 | 213 |
| **T→A** | 0.97 | 15 | 16 |
| **T→G** | 1.6 | 13 | 11 |
| **C→A** | 6.1 | 77 | 92 |
| **C→G** | 1.9 | 4.8 | 8 |
| **other** | 0.32 | 2.1 | 1.8 |
| **Total** | 16.4 | 2,129 | 1,937 |

**Table S3. Genome-wide T>C mutation rates at trinucleotide sequences in human Δ*PMS2* and Δ*MSH2* iPSCs**

| **Trinucleotide** | **Mutation rate per cell division** | | **p value** |
| --- | --- | --- | --- |
|  | **Δ*PMS2* iPSCs** | **Δ*MSH2* iPSCs** |  |
| 5’-ATC-3’ | 2.2 ± 0.5 | 1.4 ± 0.3 | 0.0444 |
| 5’-ATG-3’ | 5.9 ± 0.9 | 3.6 ± 0.7 | 0.0145 |
| 5’-CTA-3’ | 3.4 ± 0.2 | 2.3 ± 0.7 | 0.0287 |
| 5’-CTC-3’ | 2.6 ± 0.2 | 1.6 ± 0.3 | 0.0048 |
| 5’-CTG-3’ | 6.2 ± 0.8 | 4.1 ± 0.9 | 0.0205 |
| 5’-CTT-3’ | 2.3 ± 0.3 | 1.5 ± 0.5 | 0.0421 |
| 5’-GTA-3’ | 3.0 ± 0.4 | 1.6 ± 0.2 | 0.0032 |
| 5’-TTA-3’ | 3.2 ± 0.4 | 2.1 ± 0.6 | 0.0286 |
| 5’-TTC-3’ | 2.7 ± 0.3 | 1.7 ± 0.6 | 0.0325 |
| 5’-TTT-3’ | 2.5 ± 0.2 | 1.4 ± 0.4 | 0.0041 |

There were no statistical differences between the mutation rates in human Δ*PMS2* iPSCs and the corresponding mutation rates in Δ*MSH2* iPSCs at 5’-ATT-3’, 5’-GTC-3’, 5’-GTG-3’, 5’-GTT-3’, and 5’-TTG-3’ trinucleotide sequences. The genome-wide mutations and mutated sequences are from a previous study (1).

**Supplemental Reference**

1. Zou, X., Koh, G. C. C., Nanda, A. S., Degasperi, A., Urgo, K., Roumeliotis, T. I., Agu, C. A., Badja, C., Momen, S., Young, J., Amarante, T. D., Side, L., Brice, G., Perez-Alonso, V., Rueda, D., Gomez, C., Bushell, W., Harris, R., Choudhary, J. S., Genomics England Research, C., Jiricny, J., Skarnes, W. C., and Nik-Zainal, S. (2021) A systematic CRISPR screen defines mutational mechanisms underpinning signatures caused by replication errors and endogenous DNA damage. *Nat Cancer* **2**, 643-657
